# Simulating the effects of different spatio-temporal fire regimes on plant metapopulation persistence in a Mediterranean-type region

**DOI:** 10.1111/j.1365-2664.2008.01539.x

**Published:** 2008-10

**Authors:** J Groeneveld, NJ Enright, Byron B Lamont

**Affiliations:** 1 School of Geography, Geology and Environmental Science, University of AucklandNew Zealand; 2 Department of Ecological Modelling, UFZ Helmholtz Centre for Environmental ResearchLeipzig, Germany; 3 School of Environmental Science, Murdoch UniversityPerth, WA 6150, Australia; 4 Centre for Ecosystem Diversity and Dynamics, Department of Environmental Biology, Curtin University of TechnologyPerth, WA 6845, Australia

**Keywords:** *Banksia hookeriana*, fire history, fire interval, local extinction, plant metapopulation, simulation model

## Abstract

1. Spatio-temporal fire regimes are likely to shift with changes in land use and climate. Such a shift in the disturbance regime has been proposed from recent reconstructions of the regional fire history in the Mediterranean-type woodlands and shrublands of Western Australia which suggest that fire was much more frequent before 1930 (local fire intervals of 3–5 years) than it is today (local fire intervals of 8–15 years).
2. To investigate the potential biodiversity consequences of such changes in fire regime for fire-killed woody species, we developed a spatial model for the serotinous shrub *Banksia hookeriana* that grows on sand dunes of the Eneabba Plain, Western Australia. We sought to identify the envelope of fire regimes under which the spatially separated populations in this species are able to persist, and whether this encompasses the fire regimes proposed by recent fire-history reconstructions.
3. We tested two fire frequency-size distribution scenarios: (1) a scenario where fire size depends on the spatial patch configuration; and (2) a scenario depending also on available fuel (time since last fire), which reduces fire size at short inter-fire intervals.
4. In scenario 1, metapopulation persistence was only likely for mean ignition intervals at the landscape scale of 6 years. In scenario 2, persistence was likely for the whole range of fire interval distributions at the landscape scale suggested by the empirical data. However, persistence was almost impossible if the mean return fire interval at the local scale (i.e. for individual dunes) is < 8 years.
5. *Synthesis and applications*. We have demonstrated that this plant metapopulation can potentially persist over a wide range of temporal fire regimes at the landscape scale, so long as there are buffering mechanisms at work (e.g. feedback between fire spread and vegetation age) which reduces the probability of large fires at short intervals. Our findings demonstrate that at least some parts of the landscape must burn substantially less frequently on average than suggested by the empirical fire reconstructions for the early and pre-European period if populations of fire-killed woody species such as *B. hookeriana* are to be conserved.

## Introduction

Fire-prone Mediterranean-type ecosystems are plant species biodiversity hotspots and show a high level of endemism (Myers *et al.* 2000). Temporal and spatial variability of the fire regime and associated heterogeneity in regeneration conditions after fire have been identified as crucial for species coexistence (Chesson & Warner 1981; Jeltsch *et al.* 1998; Groeneveld *et al.* 2002). However, natural disturbance regimes change through time due to shifts in climate and land use, threatening the local persistence of some plant species (Hobbs & Huenneke 1992; Perry & Enright 2002; Pausas 2006). Thus, it is important to understand whether species are resilient to shifts in the disturbance regime, and what mechanisms might buffer them against the consequences of changes in environmental conditions (Grimm *et al.* 2005).

In the Mediterranean-type climate region of Western Australia, the fire regime is strongly influenced by humans and may have changed considerably during the last century as a consequence of the displacement of Aboriginal peoples and their traditional use of fire with new fire regimes following European settlement (Ward, Lamont & Burrows 2001; Abbott 2003). Previous modelling studies have investigated the conditions under which selected species can coexist in relation to fire (Bradstock *et al.* 1998; Groeneveld *et al.* 2002). However, most studies ignore the regional scale spatial dynamics of these fire-prone systems. Local populations may go extinct if fire frequency is too high, or if fire does not recur within the lifespan of the individuals (Enright *et al.* 1998), but the species can persist in the landscape if recolonization of empty (or lost) patches is possible from surviving populations elsewhere. If such spatially separated populations are linked by immigration (or recolonization), then the local populations can behave potentially as one metapopulation (Levins 1969).

The majority of reported metapopulation studies are for animal populations (Hanski 1999), since it is often difficult to determine if the assumptions of a metapopulation apply for spatially separated plant populations (Freckleton & Watkinson 2002). Freckleton & Watkinson (2002) define the following conditions for a plant metapopulation:

1. Suitable habitat occurs in discrete patches:*Banksia hookeriana* exists in at least part of its range as local populations on sand dunes that are geographically separated by uninhabitable intervening lowlands (swales) of several hundreds of metres or more in width (Calviño-Cancela, He & Lamont 2008).
2. All local populations must have a measurable risk of extinction: for fire-prone systems with fire-killed species short fire intervals can cause local extinction (Zedler, Gautier & McMaster 1983). *B. hookeriana* does not set seeds reliably until at least 5 years old, and fires at < 7-year intervals may lead to its local extinction (Enright, Lamont & Marsula 1996; Enright *et al.* 1998; Lamont *et al.* 2001). The lack of a soil seed bank in serotinous species such as *B. hookeriana* also facilitates the identification of local extinction.
3. Recolonization must be possible: genetic marker studies in *B. hookeriana* show establishment from seeds dispersed between patches (He *et al.* 2004).
4. Local populations do not have completely synchronous local dynamics: in fire-prone systems, fires generally do not burn all patches and therefore the age of the cohorts and number of stored seeds differs between patches.

*Banksia hookeriana* therefore provides a rare opportunity to examine the metapopulation dynamics of a perennial plant species in a natural landscape context where fire is the major form of landscape-level disturbance.

Metapopulation studies generally assume only two possible states for a local patch: occupied or empty (Hanski 1999). However, the demographic state of occupied patches can be out of phase (i.e. may vary in age, population density or other attributes) and these phase differences can influence the dynamic behaviour of the metapopulation (Frank & Wissel 1998; Higgins & Cain 2002). Extinction may result either from the slow decline of numbers without rescue from adjacent patches, or as a result of episodic catastrophes that affect one or more patches. The likelihood that more than one patch will be affected by a catastrophe generally includes some level of spatial correlation since neighbouring patches are more likely to suffer from any synchronous event, such as disease, storm or fire, than are widely separated patches (Pickett & White 1985).

Fire-prone environments such as Mediterranean-climate sclerophyll shrublands are characterized by frequent fire, and the species that inhabit them by attributes that facilitate either survival of extant individuals through fire, or recruitment of new individuals after fire (Gill 1981). The spatial correlation of fire among habitat patches is vital for population dynamics since fire not only causes local plant death (and possibly local extinction) but also triggers seed release (in serotinous – canopy seed stored – species) and germination (in soil seedbank species) and therefore recolonization (Lamont *et al.* 1991). Thus, consideration of spatial correlation of local extinction events alone, as in most metapopulation models (Grimm *et al.* 2004), is not sufficient to explore fire-prone plant metapopulation dynamics.

Recent fire-history reconstructions based on stem analysis of grasstrees *Xanthorrhoea* spp. suggest that high fire frequencies (fire return interval at individual grass tree locations of 3–5 years) historically were a feature of this landscape (Ward, Lamont & Burrows 2001; Colangelo *et al.* 2002; Lamont *et al.* 2003), so that local extinctions of *B. hookeriana* and other fire-killed shrubs may have been common. Genetic analyses of *B. hookeriana* seedlings have revealed evidence of long-distance post-fire dispersal of seeds between dunes up to 2·5 km apart, with rates of successful colonization of up to 7% (He *et al.* 2004). Demographic and genetic data available for this fire-sensitive perennial species provides an ideal opportunity to investigate the conservation consequences of fire regime change on metapopulation dynamics, and to further evaluate the likely validity of the ‘frequent-fire’ history proposed for plant communities of SW Australia in the pre-European period by Ward, Lamont & Burrows (2001) and others based on the grasstree record.

We constructed and analysed a patch model that simulated fire size, local population dynamics and colonization to address the following questions:

1. Under what spatial and temporal fire regimes can *B. hookeriana* persist?
2. How does the feedback between biomass accumulation (patch age) and ignition probability affect persistence?
3. What are the implications of the model results for biodiversity conservation of fire-killed woody species, and for the validity of the recently proposed ‘frequent-fire’ history?

## Methods

### study site and study species

*Banksia hookeriana* Meissner (Proteaceae) is a local endemic shrub up to 2·5 m tall confined to the upper slopes and crests of deep sand dunes of the Eneabba Plain, Western Australia, 250–330 km north of Perth (Taylor & Hopper 1988). Almost exclusively outcrossed seeds (Barrett *et al.* 2005) are stored in closed woody fruits in the plant crown for up to 12 years in a state of enforced dormancy (serotiny) and their general release is stimulated by fire (Lamont *et al.* 1991). Seeds must germinate during the subsequent wet season or perish (Enright & Lamont 1989).

### model description

The model description follows the ODD (Overview, Design concepts, and Details) protocol suggested by Grimm *et al.* (2006).

#### Overview

##### Purpose

To investigate the persistence of a plant metapopulation of a fire-killed, serotinous shrub species, *B. hookeriana*, for a wide range of spatial and temporal fire regimes. For some scenarios, not all assumptions of a true metapopulation were fulfilled (e.g. where large fires synchronize patch states or interchange of seeds is very frequent). However, given the empirical evidence of substantial genetic differentiation between local populations (He *et al.* 2004), we use metapopulation terminology throughout.

##### State variables and scales

The simulated system consists of a network of 39 patches in an area of 4 × 4 km. Information about the number of occupied patches and the temporal and spatial fire regime describe the network state. Patches are characterized by their coordinates, patch size *A_i_*, and by two state variables: number of individuals *N_i_*, and (local) time since last fire *t_j_*.

##### Process overview and scheduling

The model proceeds in time steps of fire intervals measured in years. Within each time step (fire interval), four modules are processed in the following order: determination of the time between two ignitions, fire size, local population dynamics, and long-distance seed dispersal.

#### Design concepts

##### Emergence

Local populations are coupled by seed dispersal and disturbance. The interplay of local extinctions and colonizations of different local populations results in emergent dynamics of the metapopulation.

##### Stochasticity

Stochasticity is considered in the processes: fire return interval, fire size, inter-fire survival, seed dispersal (for details see section Submodels).

##### Observations

Persistence probability *p_n_* is the main output measure and identifies the probability that the metapopulation will persist through a specified number *n* of fire events.

#### Details

##### Initialization

All simulations are initialized with all patches occupied. Every patch has recently burnt and there is no seed dispersal outside the local patch. The size of the pre-fire populations is drawn from a uniform distribution with range 1–5816 (see General model structure). Each individual disperses 235 seeds, representing the mean seed bank size for a 12 year old plant (Enright, Lamont & Marsula 1996).

##### Input

All patches are assumed to have the same habitat conditions for *B. hookeriana*.

##### General model structure

The study site is modelled as a network of 39 patches of equal size (~7·06 ha) with spatial configuration of patches described by a distance matrix (measured from centre to centre). The nature of the intervening (unsuitable) habitat and the shape of the patches are not explicitly considered. We assume that all patches have the same carrying capacity based on a maximum adult density of ~823 ha^−1^ (He *et al.* 2004).

Patches can differ in the time since last fire and therefore in age of the *B. hookeriana* cohort, and interact via seed dispersal and the spread of fire (see section Submodels for details). The time step in the model is the time between two fires, so that time steps differ in the number of years that they represent.

#### Submodels

##### Determination of fire interval at the landscape level

The time between two fires (fire interval) in the landscape (independent of their size) is determined by a two-parameter Weibull distribution (r Development Core Team 2006), where *a* is the shape parameter and *b* is the scale parameter.


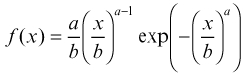


Weibull distributions were fitted to grasstree fire-history data (Enright, Lamont & Miller 2005) for a site on the Eneabba sandplain for two time-periods using the maximum likelihood r function (fitdistr, R Development Core Team 2006): one before 1930 (representing the frequent fire period) and one containing all fire events thereafter (representing the European impact period). Fire interval drawn from the Weibull distribution was increased by one to avoid fire intervals < 1, which would mean two fires in 1 year [therefore, the mean of the Weibull distribution changes to µ = *a* · Γ(1 + 1/*b*) + 1].

##### Determination of fire size

**Fig. 1:**
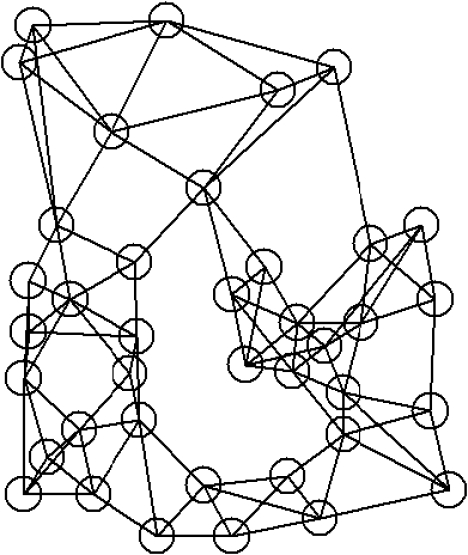
The distribution of habitat patches for *B. hookeriana*, and the fire spread network. If one patch (circle) is ignited, fire can spread to successive sets of (four) nearest neighbours (connected by lines), fire spread probability declining exponentially with distance, and depending on the scenario, fire spread probability increases with fuel age (see text for details).

We incorporated simple aggregated rules of fire spread to introduce variation of fire size and spatial correlation of fire into the modelled patch network (Fig. 1). At the time of each fire, one of the 39 patches is ignited at random. The fire then either self-extinguishes or spreads with a certain probability to the next four nearest patches, continuing to spread in this way until it self-extinguishes or has burned all patches in the network (Fig. 1). Using the four nearest neighbouring patches for the fire size determination results in some unidirectional links between patches, producing spatial variability of the fire impact, which might be crucial for allowing persistence under high frequency fire regimes. We investigated two scenarios of fire size distribution:

**1.**Fire size based on distance-dependent fire spread (i.e. whether fire will spread from patch *i* to patch *j* is determined in a Bernoulli trial using fire spread probability *ρ*), which decreases with inter-patch distance *d_i,j_* (centre to centre)


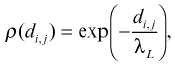


where λ_*L*_ (landscape connectivity) parameterizes the decline of the exponential function.

**2.** A fuel load dependent fire spread with fire spread probability *ρ* adjusted as follows:


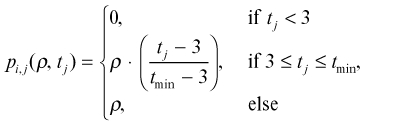


where *t_j_* is the time since last fire in target patch *j* and *t*_min_ is the stand age up to which fire spread probability increases (*t*_min_ = 12 years) as fuel load increases (i.e. ignition is impossible until time since last fire in the target patch *t_j_* exceeds 3 years) and reaches the distance-dependent fire spread probability ρ once time since last fire is 12 years.

These two fire spread scenarios are based on different assumptions concerning the major factors governing fire propagation in Mediterranean-type shrublands, each of which has some empirical support. The first assumes that severe fires are possible within a few years of previous fires under extreme weather conditions, with no clear link to fuel age (Keeley 2002; Moritz *et al.* 2004). The second assumes a relationship between fuel load and fire hazard such that the probability of a large, severe fire increases (initially at least) with increasing time since last fire as live and dead fuel loads accumulate (Minnich 1983; Loehle 2004; Piñol, Castellnou & Beven 2007).

##### Local population dynamics

If a patch burns, all individuals die and release their stored seeds (*n_t_*) depending on the time since last fire at the patch level *t* (Enright *et al*. 1996, 1998; Groeneveld *et al.* 2002; see Table 1). The number of individuals that survives until the next fire is based on the initial number of viable seeds dispersed to the patch after the last fire using a cumulative recruitment and survival probability *ρ*_*S*_ based on the geometric average of rainfall-specific survival rates from previous studies (Enright *et al*. 1996, 1998). Annual survival *s* increases up to a maximum annual survival probability once plants are 9 years old (Table 1). After 25 years, survival probability decreases by 0·01 per year reflecting a slow increase in senescence-related mortality (senescence and fire recurrence rarely allowing survivorship beyond 40 years). If more than 5000 seeds are present in a patch after a fire, we calculate the mean number of survivors as:

**Table 1:** Details of the 12 ‘Model’ scenarios tested: ‘Short’ notation for each model is determined by parameter values used to describe the ‘Dispersal mode’: local (L), long distance (LD) and directed (D), whether fire spread is a function of distance (NFB) or of distance plus ‘Biomass’ accumulation over time (FB), and the ‘Landscape connectivity’ (λ_*L*_ = 1·125 km or *λ_L_* = 4·5 km). All models have been simulated over 500 fires and 500 replicates. ‘Persistence fraction’ is the fraction of fire regimes (out of 500) where the persistence probability *p*_500_ ≥ 0·5

Model	Short	Dispersal mode	Biomass	Landscape connectivity (km)	Average fraction of patches burnt	Persistence fraction
1	LFB45	L	FB	4·5	0·73	0·72
2	LFB1125	L	FB	1·125	0·41	0·15
3	LNFB45	L	NFB	4·5	0·95	0·39
4	LNFB1125	L	NFB	1·125	0·52	0·05
5	LDFB45	LD	FB	4·5	0·73	0·95
6	LDFB1125	LD	FB	1·125	0·41	0·63
7	LDNFB45	LD	NFB	4·5	0·95	0·45
8	LDNFB1125	LD	NFB	1·125	0·52	0·31
9	DFB45	D	FB	4·5	0·73	0·95
10	DFB1125	D	FB	1·125	0·41	0·69
11	DNFB45	D	NFB	4·5	0·95	0·46
12	DNFB1125	D	NFB	1·125	0·52	0·48

**Table 2:** Model parameters

Parameter	Description (unit)	Value(s)
*n_t_*	No. of stored seeds *t* years after fire (seeds/individual)	*n*_*t*=1–5_ = 0
		*n*_*t*=6–25_ = (10, 29, 57, 92, 134, 182, 235, 293, 354, 418, 474, 522, 562, 594, 620, 640, 654, 664, 670, 674)
		*n*_*t*=26–100_ = 676
*s_t_*	Annual survival probability in year *t* (−)	*s*_*t*=1–8_ = (0·031, 0·61, 0·817, 0·886, 0·919, 0·938, 0·95, 0·959)
		s_*t*=9–25_ = 0·977
*t_s_*	age of increased mortality (years)	*t_s_* = 25
*m*	annual decrease in survival probability due to senescence (−)	*m* = 0·01
*a*	maximum longevity (years)	*a* = 100
*K*	Capacity (individuals)	*K* = 5816
λ	Mean dispersal distance (LDD) (km)	λ = 1·1
*r*_0_	Patch radius (km)	*r*_0_ = 0·15
*t*_min_	Vegetation age of maximum fire spread (years)	*t*_min_ = 12
*b*	Fraction of LDD seeds (−)	*b* = 0·1

*N*_Sur_(*t*) = min(*K*, *N*_0_·*ρ*_*S*_),

where *K* is the capacity of the patch. The number of surviving plants at age *t* is the product of the initial number of seeds *N*_0_ and the cumulative survival probability *ρ*_*S*_ to reach age *t*(rounding of *N_sur_* is done stochastically). If the initial seed number *N*_0_ is below 5000, we draw the number of surviving plants *N*_Su*r*_ from a binomial distribution with a probability of *ρ*_*s*_ and *N*_0_ tries.

##### Long-distance seed dispersal and colonization

If occupied patches burn, then their seeds can potentially colonize empty patches by long-distance dispersal (LDD). Most released seeds remain in the patch itself (90%) and only a fraction (10%) will be dispersed outside the patch. The probability of dispersing a seed into a ring (annulus) at distance *d*is assumed to decrease exponentially, and we approximate the probability *ρ*_*ij*_ to disperse a seed from patch *i* to patch *j* by LDD as:


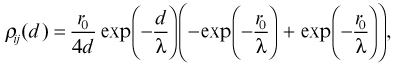


where *d* is the distance between patch *i* and *j* (centre to centre), *r*_0_ is the patch radius (assuming that all patches are circles of the same size), and λ parameterizes the exponential decay. For the simulation experiments, we used three dispersal modes: local dispersal (all seeds remain in the patch where they were produced), LDD (mean dispersal distance λ = 1·1 km), and directed dispersal (the fraction of long-distance dispersed seeds are dispersed equally between all other burnt patches). We assume that only burnt patches can be colonized, since unburnt patches are occupied by extant individuals and competition among established plants makes successful recruitment from seeds unlikely (Enright *et al.* 1998). Finally, we approximated the number of seeds that arrive at patch *i* dispersed from patch *j* by a binomial distribution if the number of dispersed seeds is below 5000; otherwise, we use the average (see process Local population dynamics for details).

### simulation experiments

We conducted 12 simulation experiments (Table 1) representing two fire size scenarios (FB: feedback between fire spread and fuel load; NFB: no feedback), two connectivities of the landscape for fire spread, λ_*L*_[λ_L_ = (4·5, 1·125) km]– which results in an average fraction of burnt patches AF [AF = (95, 52)%] assuming NFB, and three dispersal modes (local, LDD and directed). We varied the scale (1–25 years) and shape parameters (1–10·5) of the landscape fire interval Weibull distribution systematically in all simulation experiments. Each simulation experiment is named after its configuration (Table 1).

For all simulation experiments and parameterizations, the model ran for 500 time steps (i.e. 500 fires and a minimum of 500 years) for each of 500 replicate runs. Results are presented for each scenario as the persistence probability *p*_500_ that populations could persist through 500 fire events.

## Results

**Fig. 2:**
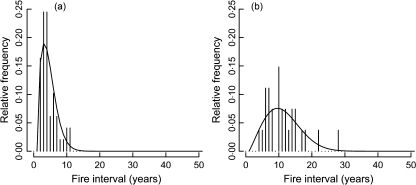
Maximum-likelihood estimates for fire interval distributions (a) before 1930, scale = 4 and shape = 1·6, and (b) after 1930, scale = 11·7 and shape = 2·1 based on grasstree fire-history data for shrublands near Eneabba, Western Australia. Bars indicate measured fire intervals from black bands on stems of grasstrees (Enright *et al.* 2005) and lines are the estimated fire interval Weibull distributions.

Weibull distribution scale and shape parameters differed markedly for the fire interval distributions before and after 1930 based on the grasstree fire-history record (Fig. 2). The mean of the estimated fire interval distribution before 1930 was shorter (*µ* = 4·6 years) and less variable (SD = 2·3 years) than for the period after 1930 (*µ* = 11·4 years, SD = 5·2 years).

**Fig. 3:**
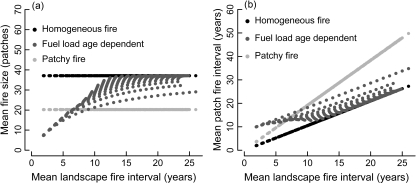
Relationship between mean landscape (global) fire interval, generated by Weibull distributions of different mean and variance, and average fire size (a) and (b) mean patch (local level) fire interval. When fire risk declines exponentially with distance between patches the relationship is linear (light grey and black dots). If fire risk also depends on vegetation age (fuel load), the relationship is more complex (dark grey dots).

Mean fire size differed among simulations based on the value for landscape connectivity λ_*L*_: for NFB (distance-dependent fire-spread) with λ_*L*_ = 4·5 km, 95% of all patches burn on average, so that fires tend to be uniformly large, whereas with λ_*L*_ = 1·125 km, only 52% of the landscape burns on average resulting in a multimodal fire size distribution, so that fires are smaller and more patchy (Fig. 3a). The relationship between mean fire interval at the landscape and local (patch) levels is linear (Fig. 3), and the steepness of the trend between mean fire intervals of the different spatial scales depends on the average fraction of burnt patches (Fig. 3). For FB simulations (feedback between fuel load and ignition probability), the linear relationship between fire interval at the landscape scale and fire size disappears (Fig. 3a), as does that between fire interval at the landscape and the local (patch) scale (Fig. 3b).

**Fig. 4:**
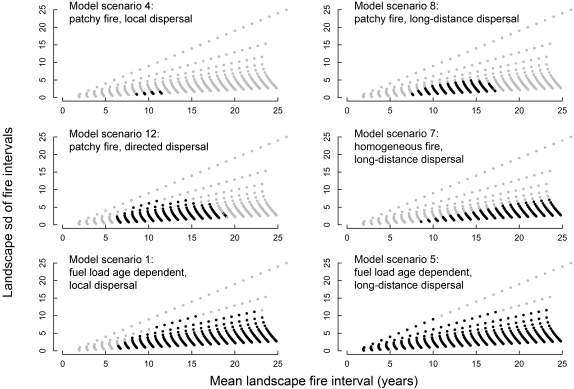
Persistence of *B. hookeriana* in relation to the mean fire interval and the standard deviation of the fire interval distribution at the landscape scale. Dark dots indicate scenarios where *B. hookeriana* persisted in at least 50% of all (500) runs. For reference, local mean fire intervals are estimated as 4·6 years for the pre-European fire regime (before 1930) and as 11·4 years after 1930.

For NFB and landscape connectivity λ_*L*_ = 4·5 km, patch states were highly synchronized due to large fire size (AF = 95%), and dispersal mode has little effect on metapopulation persistence, with local populations persisting for landscape level mean fire intervals 8·6 years (see Table 1; scenarios 3, 7, 11). Under low connectivity (*λ_L_* = 1·125 km) and local dispersal, *p*_500_ ≥ 0·5 was only possible for mean fire intervals at the landscape level between 8·6 years and 11·5 years (scenario 4, Fig. 4). LDD increased considerably the range of fire regimes for *p*_500_ ≥ 0·5 (7·5–17·2 years, scenario 8), and with directed dispersal, persistence was possible for fire intervals between 6·3 years and 19 years (scenario 12). Average local fire intervals similar to the pre-1930 empirical fire reconstruction (~5 years) required a mean landscape fire interval of approximately 2 years (scenarios 4, 8, 12), and under this fire regime, long-term persistence is hardly possible (*p*_500_ < 1%, Fig. 4).

**Fig. 5:**
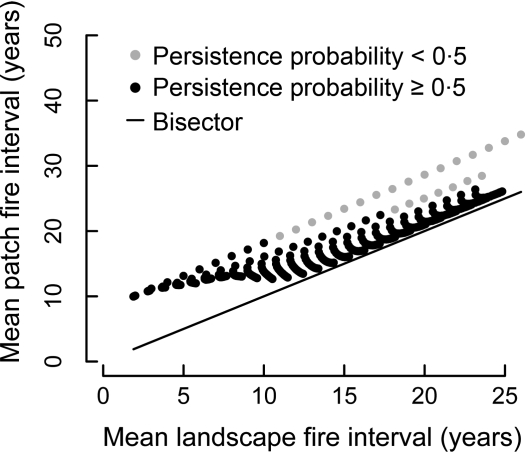
Persistence (black dots: population persisted in more than 50% of all 500 runs) in relation to mean fire intervals at the landscape and local patch levels, if fire risk is a function of fuel load and inter patch distance (LDD, λ_*L*_ = 1·1 km, model scenario LDFB45). Regardless of the mean fire interval at the landscape level, local fire interval cannot fall below 10 years on average at the patch level. For reference, local mean fire intervals are estimated as 4·6 years for the pre-European fire regime (before 1930) and as 11·4 years after 1930.

For FB with local dispersal, the metapopulation can persist over a wide range of fire intervals (≥ 6·7 years) at the landscape level (Fig. 4; scenario 1). When LDD is included, the persistence range expands over the whole spectrum of simulated mean fire intervals (i.e. ≥ 1·9 years) at the landscape level (Fig. 4, scenario 5). However, if fire intervals are short, then fires tend to be small and patchy and (local) fire return intervals never drop below a mean of 10 years (Figs 3 and 5).

Overall, the range of fire regimes for which *B. hookeriana* can persist is smallest (*p*_500_ 0·5 for 5% of all fire regimes, Table 1) where dispersal mode is local, ignition probability does not depend on fuel age and fires are patchy (scenario 4), and greatest (*p*_500_ 0·5 for 95% of all fire regimes, Table 1) where fire spread probability depends on fuel age, there is LDD and landscape connectivity for fire is high (scenarios 5 and 9).

## Discussion

Previous simulation studies have predicted that mean fire intervals must exceed 8 years if *B. hookeriana* is to persist (Enright *et al.* 1998; Lamont *et al.* 2001; Groeneveld *et al.* 2002), which contradicts recent grasstree fire-history evidence that suggests fire was much more frequent in the (pre-European) past (Ward, Lamont & Burrows 2001; Lamont, Wittkuhn & Korczynskyi 2004). Our simulations have expanded the analysis of this question to incorporate multiple local populations in the context of a metapopulation that more fully explores how spatial and temporal patterns might interact to facilitate persistence of fire-killed species in a frequent-fire environment. We show that, at the landscape scale, colonization events and feedback between spatial fire spread and the amount of combustible biomass can compensate for local extinctions due to short fire or extremely long fire intervals and therefore allow persistence even if fires occur every 2–3 years in the system – so long as individual fires burn only a small fraction of patches. However, at the local (single patch) scale our results agree with the previous findings, with persistence only expected if the local mean fire interval at the patch level is 8–10 years.

Feedback between spatial fire spread and the amount of combustible biomass buffers changes of the fire regime at the landscape level (i.e. if the whole system has burnt recently), the subsequent fires will be small, and vice versa, as also shown by Piñol, Castellnou & Beven (2007). This feedback, together with LDD, allows *B. hookeriana* to persist over a wide range of possible fire regimes at the landscape level. Successful LDD events may buffer small populations against potentially deleterious effects associated with genetic drift, inbreeding and pollen limitation, and Bossuyt (2007) has illustrated recently how genetic rescue from introduced pollen can increase viable seed set. This might be important for many species, since we are only now beginning to quantify rates of successful recruitment from long-distance dispersal events due to recent advances in molecular ecology and new modelling approaches (Nathan & Muller-Landau 2000; Cain, Milligan & Strand 2000; Tackenberg 2003; Schurr *et al.* 2005). Given the high rate of LDD measured for *B. hookeriana* in our study area (He *et al.* 2004), the genetic risks associated with small population size in this species (and those with similar life-history attributes) may be quite low so long as fragments are no more than a few kilometres apart. Indeed, He *et al.* (2004) found genetic diversity to be high, with no relationship between genetic diversity and population size.

Long-range dispersal is not only important in compensating for local extinction due to short fire events, but also for long fire intervals. For example, Menges & Hawkes (1998) found that population size for a number of herbaceous species in Florida pine scrub was dependent upon the spatial distribution and extent of open sites, and the time for which they were available, following fire. Species behaved as metapopulations, with population size and number likely to decrease if fire interval increased due to the loss of open sites and increased distance between remaining open-habitat fragments. In the present context, this is important for conservation management planning since longer mean inter-fire intervals at the patch level may result from fragmentation and reduced connectivity of the landscape due to ongoing human impacts associated with land clearing for roads, agriculture, urbanization and other infrastructure.

In relation to more frequent fire, our findings do not support recent reconstructions of fire histories for SW Australia using grasstrees (Ward, Lamont & Burrows 2001; Lamont *et al*. 2003, 2004), which reveal an apparent mean fire interval of only 3–5 years in the period 1750–1930. These authors argue that this represents the ‘Aboriginal’ fire regime for the region and may have been in place for thousands of years prior to European settlement of Australia. Historical accounts of Aboriginal use of fire suggest that they burned vegetation frequently, but in small patches (Abbott 2003). While at first glance our findings seem to lend support to the ‘Aboriginal’ fire regime hypothesis, they do not. The Aboriginal fire regime scenario implies that all of the landscape is burned within a period of 3–5 years by fires of unknown (but suggested small) size. Since individual grasstree stems show regular (apparently fire-induced) dark bands at these intervals prior 1930, this means that fire has burned the same patches of vegetation (or at least the same grasstrees) repeatedly at this frequency. This contradicts our findings that average local scale (patch) fire interval must exceed 8–10 years for persistence to be reasonably likely.

The frequent fire scenario tested here can only be rationalized with our results if fires consistently spare parts of the landscape, which therefore have a longer local fire return interval. Fire could occur somewhere in the landscape (here modelled on an area of 4 × 4 km) every year, but can only affect a small number of patches per fire if persistence is to be possible. Since our previous studies have shown that interspecific competition and annual variability in growing conditions (factors not considered here) further reduce overall rates of survivorship and seed production (Lamont *et al.* 2001; Groeneveld *et al.* 2002), the minimum mean fire interval compatible with persistence of *B. hookeriana* is most likely rather higher than 10 years, making the results reported here a conservative underestimate.

## Conclusions

A patch model describing the dynamics of the SW Australian fire-killed perennial shrub, *B. hookeriana*, in relation to fire regime is presented. Assuming that fire spread is a function of inter-patch distance and the amount of available biomass (fuel load), spatially separated populations of *B. hookeriana* can persist for a wide range of possible fire regimes at the landscape level. The feedback between fire spread probability and fuel load (stand age) buffers the effects of variable temporal fire regimes at the landscape level. Long-distance seed dispersal is crucial to population persistence for short mean fire intervals at the landscape level, as suggested by recent empirical data. Our study indicates that *B. hookeriana* is highly robust in terms of changes to the overall fire regime. However, our findings do not support the recently reported ‘frequent-fire history’ for sclerophyll shrublands in SW Australia. Without refuges where fire occurs less frequently, the frequent-fire history would lead in the long term to the local extinction of this fire-killed perennial species. The same fate would be true for many other fire-sensitive species in the region, suggesting that such a pre-European fire regime is not compatible with the persistence of many fire-killed perennial plant species in the SW Australian flora. These results cast further doubt over whether the grasstree fire-history record can be used to reconstruct the fire regime (Miller *et al*. 2007) and suggest that caution is needed in any attempt to frame future fire management policies around such reconstructed fire regimes.
